# Analyzing the ecological relations of technology innovation of the Chinese high-tech industry based on the Lotka-Volterra model

**DOI:** 10.1371/journal.pone.0267033

**Published:** 2022-05-31

**Authors:** Ming Xia, Xiangwu He, Hui Lin, Zhimin Xie, Yubin Zhou

**Affiliations:** 1 School of Economics and Management, Tongji University, Shanghai, China; 2 Business School, Jiaxing University, Jiaxing, China; Institute for Advanced Sustainability Studies, GERMANY

## Abstract

Technology innovation has become an important driving force of economic and social development and has received wide attention from academics. Most scholars mainly take technology innovation as an overall variable to explore its impact on the economy and society. The main contribution of this study is to open the black box of technology innovation and introduce the lotka-Volterra model to explore the internal structure of technology innovation in the Chinese high-tech industry and to analyze the ecological relationships, evolutionary trends, equilibrium states of six technology innovation species including independent innovation (II), technology import (TI), research & development (RD), technology renovation (TR), foreign technology acquisition (FTA), and domestic technology purchase (DTP). The results of the study show that, First, the ecological relationship between prey and predator is observed between RD and TR, DTP and FTA, and II and TI. Second, no equilibrium state is observed between TD and TF and II and TI. Third, an unstable equilibrium state is observed between RD and TR.

## 1 Introduction

In the context of the COVID-19 pandemic, technology innovation plays a very important role in economic and social development as well as coping with global emergencies [1]. How to carry out technology innovation more effectively has attracted extensive attention from scholars [2]. High-tech industries, which are knowledge and technology-intensive, have long been the main carrier and driving force of technology innovation in China [3]. Based on the subjective desire for industrial upgrading or the objective need of the market, technology innovation has become an important driving force for the transformation and upgrading of high-tech industries and the enhancement of their core competitiveness [4]. The sources of technology innovation in high-tech industries include both internal and external aspects [5]. Internal technology sources include independent research and development (RD) and technological renovation (TR) [6, 7], while external technology sources include foreign technology acquisition (FTA) and domestic technology purchase (DTP) [8, 9]. The ecological relationships of technology innovation among RD, TR, FT, and DT not only affect the efficiency of technology innovation output of high-tech industries, but also have a great impact on the innovation ability, transformation, and upgrading of high-tech industries. Correctly analyzing and comparing the ecological relationships of the technology innovation in China’s high-tech industry has become a real challenge that hinders the healthy and sustainable development of this industry.

Most of the existing studies have focused on analyzing technology innovation as a black box-like variable from two perspectives. On the one hand, some scholars have focused more on the role of technology innovation in the economic system. Jian et al. (2010) established the interaction between network relationships, trust, knowledge sharing, and technology innovation performance based on structural equation modeling [10]. Jiang et al. (2012) argued that high-quality technology innovation can promote technology standards and the widespread implementation of high-level technology standards can promote technology innovation [11]. Iranmanesh (2014) discussed the antecedents and outcomes of green technology innovation adoption in Malaysian transport firms [12]. Verbano and Crema (2016) investigated the role of technology innovation strategies on intellectual capital development and hence on innovation performance of manufacturing SMEs [13]. Feng and Yuan (2016) analyzed the impact of technology innovation and spillovers on the carbon intensity of human well-being using panel data of 30 provinces in China from 2005 to 2010 [14]. On the other hand, other scholars have concentrated on the factors for technology innovation. Shi and Zhu (2014) empirically examined the relationship between age, political connections, and technology innovation outputs of the pharmaceutical industry in China [15]. Sun (2015) explored the role of government in the technology innovation process in China from the perspective of strategic entrepreneurship [16]. Guo et al. (2016) argued that four types of factors, classified as innovation policy, innovation input, innovation capacity, and innovation organization, may influence the transformation of coal resource-based economies [17].

However, as a complex concept, technology innovation consists of several components depending on the source of the technology. In recent years, studies have discussed the components of technology innovation and their interrelationships. Kim and Stewart (1993) examined the relationship between domestic R&D and technology imports [18]. Lee (1996) analyzed the relationship between technology imports and the R&D efforts of Korean manufacturing firms through a two-stage approach [19]. Yu et al. (2016) conducted a study on the relationship between technical sources and innovation output of the Chinese high-tech industry [20]. But, the study of the relationship between different types of technology innovation did not cover all types, and the internal organic structure of technology innovation deserves further exploration.

Therefore, from an analytical point of view, opening the black box of technology innovation and exploring the internal organic structure of technology innovation is conducive to analyzing the internal connections and interactions between various types of technology innovation. In terms of research methodology, the static, one-way evaluation method of the relationship between factors related to technology innovation cannot conform to the dynamic development trend presented by technology innovation [21]. It is necessary to conduct a dynamic, ecological analysis of the two-way relationships of technology innovation. The Lotka-Volterra model is the most commonly used model for analyzing population competition and is very suitable for analyzing the evolution of ecological relationships of technology innovation populations.

Based on the above analysis, this study further opens the black box of technology innovation and divides it into two parts: independent innovation (II), including research & development (RD) and technology renovation (TR); and technology imports (TIs), including foreign technology acquisition (FTA) and domestic technology purchase (DTP), explores the complex dynamic relationship between the four types of technology innovation, and constructs a theoretical model among the four populations competition. We introduce the Lotka-Volterra model to explore the complex ecological relationships among technology innovation types in Chinese high-tech industries based on the data of technology innovation in China from 1995 to 2015. This study provides a theoretical basis for optimizing the allocation of technology innovation resources in high-tech industries and improving the efficiency of technology innovation. The key questions to be addressed in this study are as follows. What is the internal structure of technology innovation? What is the ecological relationship between the four types of technology innovation? What strategies should governments and enterprises adopt to strengthen technology innovation?

The remainder of this paper is organized as follows. Section 2 provides a short review of ecological relationships and the Lotka-Volterra model, and introduces the empirical model based on the Lotka-Volterra model and describes the dataset; Section 3 provides the empirical estimates and discusses the results; Section 4 presents the concluding remarks, policy implications, and limitations of this study briefly.

## 2 Methodology

### 2.1 Ecological relationships and Lotka-Volterra model

Based on the theory of innovation ecosystems [22, 23], the six types of technology innovations are regarded as six species that constitute a technology innovation ecosystem. We adopt the definition of ecological relationships (ERs) to discuss the cooperative and competitive interactions among the technology innovation species. Therefore, it is crucial to understand and consider the ecological relationships [24] that influence the balance and dynamics of technology innovation ecosystems [25].

Currently, descriptive mathematical models have been developed to predict the spread of species innovations and population development, such as the Gompertz model [9], logistic model [26], Bass model [27], GM (1, 1), and Lotka-Volterra model [28]; however, only the Lotka-Volterra model can examine the ERs of species.

The Lotka-Volterra model, first proposed by the American ecologist Lotka and the Italian mathematician Volterra [29], was derived to explore the growth curve-based interactions between two competing species, and it is often applied to describe the growth and decline of two competing groups in an ecosystem [30]. In recent years, this model has been applied in the field of socioeconomics to examine the issues of economic growth and population control [31]. Modis (1997) applied the model to study the dynamic relationship between competitors in a finite space [32]. Fu et al. (2017) used the Lotka-Volterra model to study the relationship between rural and urban Internet users in China [33]. Chakrabarti (2016) applied the Lotka-Volterra equations to model the evolution of the technology frontier [34]. Cordes and Schwesinger (2014) used an extended Lotka-Volterra model to capture the competitive interactions between two technologies [35]. Zhang and Lam (2013) introduced the Lotka-Volterra model to study the interaction between maritime sectors [36]. Lin (2013) applied the Lotka-Volterra model to analyze the competitive relationship between mobile cellular broadband and fixed broadband [37]. This study assumes that the technology innovation ecosystem conforms to the fundamental Lotka–Volterra model and that the ecological relationships can be described by corresponding mathematical equations.

### 2.2 Model analysis

According to the literature [31, 37, 38], the ecological relationships between two varieties of technology innovation in China’s high-tech industry can be described by the Lotka–Volterra model using Eqs (1) and (2):

$$\frac{dX_{1}(t)}{dt}=f_{1}(X_{1},X_{2})=X_{1}(t)(\alpha_{10}+\alpha_{11}X_{1}(t)+\alpha_{12}X_{2}(t))=\alpha_{10}X_{1}(t)+\alpha_{11}X_{1}(t)X_{1}(t)+\alpha_{12}X_{2}(t)X_{1}(t),$$ (1)

$$\frac{dX_{2}(t)}{dt}=f_{2}(X_{1},X_{2})=X_{2}(t)(\alpha_{20}+\alpha_{21}X_{1}(t)+\alpha_{22}X_{2}(t))=\alpha_{20}X_{2}(t)+\alpha_{21}X_{1}(t)X_{2}(t)+\alpha_{22}X_{2}(t)X_{2}(t).$$ (2)

In Eqs (1) and (2), *X*_1_ and *X*_2_ represent two technology innovation species; *dX*_1_(*t*)/*dt* and *dX*_2_(*t*)/*dt* denote the annual scale of *X*_1_ and *X*_2_ at each year t, respectively; *X*_1_(*t*) and *X*_2_(*t*) are the cumulative scales of *X*_1_ and *X*_2_ up to year t, respectively; *X*_1_(*t*)*X*_1_(*t*) and *X*_2_(*t*)*X*_2_(*t*) refer to the same technology innovation species interacting with itself; *X*_1_(*t*)*X*_2_(*t*) and *X*_2_(t)*X*_1_(*t*) denote two technology innovation species interacting with each other; *α*_*ij*_ is used to determine the competition and cooperation of two technology innovation species; *α*_10_ and *α*_20_ are the logistic parameters representing the intrinsic growth rates of *X*_1_(*t*) and *X*_2_(*t*), respectively; *α*_11_ and *α*_22_ are the limitation parameters of the niche capacity for *X*_1_(*t*) and *X*_2_(*t*), respectively; and considering the possible endogeneity between variables, *α*_12_ and *α*_21_ are the interaction parameters representing how two technology innovation species affect each other. Modis (1999) summarized the possible types of ecological relationships of two species according to the signs of parameters *α*_12_ and *α*_21_ [39], as shown in Table 1.

**Table 1:** Ecological relation types according to the sign of *α*_12_ and *α*_21_.

*α*_12_	*α*_21_	Type	Explanation
−	−	Pure competition	Both species suffer from each other’s existence
+	+	Mutualism	Symbiosis or a win-win situation
+	−	*X*_1_ as predator and *X*_2_ as prey	*X*_2_ serves as direct food for *X*_1_
−	+	*X*_1_ as prey and *X*_2_ as Predator	*X*_1_ serves as direct food for *X*_2_
−	0	Amensalism	One suffers from the existence of the other, while the latter is impervious
+	0	Commensalism	One benefits from the existence of the other, while the latter remains unaffected
0	0	Neutralism	No interaction

### 2.3 Discretization

Since Eqs (1) and (2) are continuous-time models and the technology innovation scale in this study uses discrete data, the continuous Lotka–Volterra model must be converted into a discrete-time version. Currently, there are three discretization methods for the Lotka-Volterra model: the Leslie method, the log-integral method, and the gray method. In this study, the Leslie method was used for discretization, and the other two methods (See S1 Appendix for the specific formula derivation) were subsequently used for robustness testing.

According to the Leslie method [40], Eqs (1) and (2) can be transformed into discrete Eqs (3) and (4):

$$X_{1}(t+1)=\frac{b_{10}X_{1}(t)}{1-b_{21}X_{1}(t)-b_{12}X_{2}(t)},$$ (3)

$$X_{2}(t+1)=\frac{b_{20}X_{2}(t)}{1-b_{21}X_{1}(t)-b_{22}X_{2}(t)}.$$ (4)

In Eqs (3) and (4), *b*_*ii*_ and *b*_*i0*_ are logistic parameters when only one species *i* lives alone and *b*_*12*_ and *b*_*21*_ refer to the degree of the effect of the relationship between two species on their respective growth rates. The relationship between the parameters in Eqs (1) and (2) and those in Eqs (3) and (4) are denoted as Eq (5). Note that the sign of *α*_*ij*_ is the same as that of *b*_*ij*_:

$$\alpha_{i0}=\ln\;b_{i0},\;\alpha_{ij=}\frac{b_{ij}\;\ln\;b_{i0}}{b_{i0}-1},\;i,j=1,2.$$ (5)

To perform regression calculations, Eqs (3) and (4)) can be transformed into Eqs (6) and (7):

$$\frac{X_{1}(t)}{X_{1}(t+1)}=\frac{1}{b_{10}}-\frac{b_{11}}{b_{10}}X_{1}(t)-\frac{b_{12}}{b_{10}}X_{2}(t),$$ (6)

$$\frac{X_{2}(t)}{X_{2}(t+1)}=\frac{1}{b_{20}}-\frac{b_{21}}{b_{20}}X_{1}(t)-\frac{b_{22}}{b_{20}}X_{2}(t).$$ (7)

Let

$$Y_{i}(t)=\frac{X_{i}(t)}{X_{i}(t+1)},c_{i0}=\frac{1}{b_{i0}},c_{i1}=-\frac{b_{i1}}{b_{i0}},c_{i0}=-\frac{b_{i2}}{b_{i0}},i=1,2.$$ (8)

Eqs (6) and (7): can be converted into Eqs (9) and (10):

$$Y_{1}(t+1)=c_{10}+c_{11}X_{1}(t)+c_{12}X_{2}(t),$$ (9)

$$Y_{2}(t+1)=c_{20}+c_{21}X_{1}(t)+c_{22}X_{2}(t).$$ (10)

Using the linear least square method, we can estimate the parameters in Eqs (8) and (10).

### 2.4 Model prediction ability calculation

This study uses the percentage error (PE), mean absolute percentage error (MAPE), mean absolute error (MAE), and root mean square error (RMSE) to measure the prediction ability of the model. PE and MAPE, MAE, and RMSE can be calculated using Eqs (11) ~ (14):

$$PE=\frac{A_{t}-P_{t}}{A_{t}}\times 100\%,$$ (11)

$$MAPE=\frac{\sum_{t=1}^{n}\left|\frac{\left(A_{t}-P_{t}\right)}{A_{t}}\right|}{n}\times 100\%,$$ (12)

$$MAE=\frac{1}{n}\sum_{t=1}^{n}|A_{t}-P_{t}|,$$ (13)

$$RMSE=\sqrt{\frac{1}{n}\sum_{t=1}^{n}{\left(A_{t}-P_{t}\right)}^{2}}.$$ (14)

In Eqs (11) ~ (14), *n* represents the number of observations, *A*_*t*_ is the actual value, and *P*_*t*_ is the predicted value of *A*_*t*_. For all the above indexes, a smaller value implies a higher prediction accuracy. According to Lewis [41], the forecast capability levels of the MAPE are shown in Table 2.

**Table 2:** Forecast ability levels of MAPE.

MAPE (%)	Prediction capability
<10	Highly accurate
10―20	Good
20―50	Reasonable
>50	Inaccurate

Source: Lewis (1982) [41].

### 2.5 Equilibrium analysis

Ecological relationships of technology innovation will reach a stable equilibrium when the volume of each species becomes constant, which means that the results of Eqs (1) and (2) are both zero [42–45]. We can obtain the equilibrium Eqs (15) and (16):

$$\frac{dX_{1}(t)}{dt}=f_{1}(X_{1},X_{2})=X_{1}(t)(\alpha_{10}+\alpha_{11}X_{1}(t)+\alpha_{12}X_{2}(t))=0,$$ (15)

$$\frac{dX_{2}(t)}{dt}=f_{2}(X_{1},X_{2})=X_{2}(t)(\alpha_{20}+\alpha_{21}X_{1}(t)+\alpha_{22}X_{2}(t))=0.$$ (16)

Because *X*_1_(*t*)>0, *X*_2_(*t*)>0, Eqs (15) and (16) can be transformed into Eqs (17) and (18):

$$L_{1}:\alpha_{10}+\alpha_{11}X_{1}(t)+\alpha_{12}X_{2}(t)=0,$$ (17)

$$L_{2}:\alpha_{20}+\alpha_{21}X_{1}(t)+\alpha_{22}X_{2}(t)=0.$$ (18)

In Eqs (17) and (18), X_1_ is the horizontal coordinate and X_2_ is the vertical coordinate. The evolutionary trend graph of X_1_ and X_2_ can be drawn in the plane rectangular coordinate system, according to which the evolutionary trends of the ecological relationships of technology innovations can be analyzed.

By solving Eqs (17) and (18), we can obtain the equilibrium points, as shown in Eq (19):

$$(\dot{x_{1},}\dot{x_{2}})=(\frac{\alpha_{10}\alpha_{22}-\alpha_{12}c_{20}}{\alpha_{12}c_{21}-\alpha_{11}c_{22}},\frac{\alpha_{11}\alpha_{20}-\alpha_{10}\alpha_{21}}{\alpha_{12}\alpha_{21}-\alpha_{11}\alpha_{22}}).$$ (19)

However, not every equilibrium point is stable under the condition of environmental change. We applied the Jacobian matrix to calculate the Jacobian eigenvalue of each group and assess which equilibrium point of each group is stable. The condition for the stability of an equilibrium point is that both real numbers of eigenvalues are negative [34]. The Jacobian matrix is expressed in Eq (20):

$$J(f_{1},f_{2})=\left|\begin{array}{cc} \frac{\partial f_{1}}{\partial X_{1}} & \frac{\partial f_{1}}{\partial X_{2}}\\ \frac{\partial f_{2}}{\partial X_{1}} & \frac{\partial f_{2}}{\partial X_{2}} \end{array}\right|=\left(\begin{array}{cc} \alpha_{10}+2\alpha_{11}X_{1}+\alpha_{12}X_{2} & \alpha_{12}X_{1}\\ \alpha_{21}X_{2} & c_{20}+\alpha_{21}X_{1}+{2\alpha }_{22}X_{2} \end{array}\right).$$ (20)

### 2.6 Variables and data

**Fig 1:**
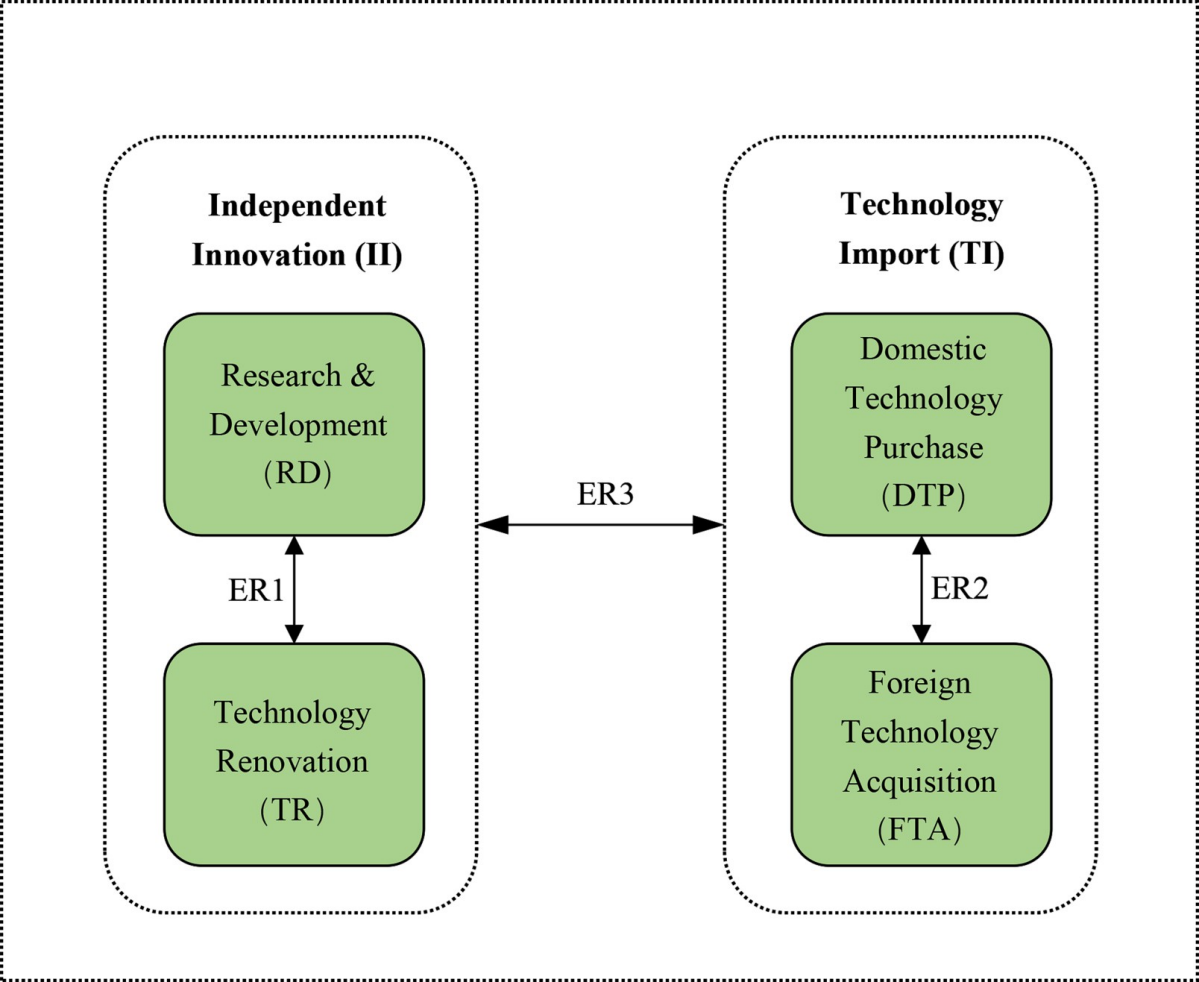
The research framework of the ecological relationships among six technology innovations.

Since the Lotka-Volterra model is more suitable for representing the relationship between two variables and two types of technology innovation are observed in the high-tech industries, namely, II, which can further be divided into RD and TR, and TI, which can further be divided into DTP and FTA, this study investigates the ecological relationships of three pairs of technology innovation: RD and TR (denoted ER1), DTP and FTA (denoted ER2), and II and TI (denoted ER3). The research framework of the ER of technology innovation is shown in Fig 1. The names, codes, units, definitions, and descriptions of all variables are shown in Table 3.

**Table 3:** Variables’ names, codes, units, definitions, and descriptions of all variables.

Variable name	Code	Units	Definitions	Description
Research & Development	RD	100 million yuan	Expenditure on Internal R&D	Refers to the actual expenditure of the unit for internal R&D activities during the reporting year.
Technology Renovation	TR	100 million yuan	Expenditure for Technical Renovation	Refers to the expenses for the technical renovation of the enterprise during the reporting period.
Domestic Technology Purchase	DTP	100 million yuan	Expenditure for the Purchase of Domestic Technology	Refers to the expenses for the purchase of scientific and technological achievements of other units in the territory during the reporting period.
Foreign Technology Acquisition	FTA	100 million yuan	Expenditure for Acquisition of Foreign Technology	Refers to the expenses incurred during the reporting period for the purchase of technologies of foreign or Hong Kong, Macao, and Taiwan and expenditure for assimilation of the technologies.
Independent Innovation	II	100 million yuan	RD plus TR	Refers to the expenditures for RD and TR.
Technology Innovation	TI	100 million yuan	TD plus TF	Refers to expenditures for TD and TF.

Data source: China statistical yearbook on the high-tech industry from 1995 to 2015.

The original data in this paper are obtained from the China Statistical Yearbook on the high-tech industry (1995~2015). Since technology innovation is a long process and requires a long time to accumulate, considering the availability of data, this study takes 2015 as the cut-off point and uses technology innovation data within 20 years as the sample. Considering the effect of the annual price level of technology innovation expenditure, the original data were deflated using the annual CPI index in 1995, and the data were obtained from the China Statistical Yearbook (1995~2015). Due to the data requirements of the Lotka-Volterra model, we also calculate the cumulative data for six variables of technology innovation in high-tech industries. The data of technology innovation in high-tech industries in China from 1995 to 2015 are shown in Table 4.

**Table 4:** Expenditures for technology innovation in the Chinese high-tech industry from 1995 to 2015.

year	Annual data (Units: 100 million)						Accumulative data (Units: 100 million)					
Var	RD	TR	DTP	FTA	II	TI	RD	TR	DTP	FTA	II	TI
1995	17.85	82.27	4.25	31.43	100.12	35.69	17.85	82.27	4.25	31.43	100.12	35.69
1996	30.96	78.45	2.43	24.86	109.41	27.28	28.58	72.43	2.24	22.95	101.01	25.19
1997	42.02	81.51	2.74	35.04	123.53	37.78	37.74	73.21	2.46	31.47	110.95	33.93
1998	56.45	68.20	2.00	24.30	124.65	26.30	51.11	61.74	1.81	22.00	112.85	23.81
1999	67.56	69.26	2.86	26.23	136.81	29.09	62.04	63.60	2.63	24.08	125.64	26.71
2000	111.04	104.75	7.21	50.41	215.79	57.62	101.55	95.79	6.59	46.11	197.34	52.70
2001	157.01	117.23	3.77	79.52	274.24	83.28	142.60	106.47	3.42	72.22	249.07	75.64
2002	186.97	152.43	5.89	98.95	339.40	104.84	171.18	139.56	5.39	90.60	310.74	95.99
2003	222.45	155.04	8.57	99.19	377.48	107.75	201.25	140.26	7.75	89.74	341.52	97.49
2004	292.13	187.90	8.57	124.37	480.04	132.93	254.38	163.62	7.46	108.29	418.00	115.76
2005	362.50	159.02	9.54	112.32	521.52	121.85	310.08	136.03	8.16	96.07	446.10	104.23
2006	456.44	171.91	10.23	89.58	628.34	99.81	384.63	144.86	8.62	75.49	529.49	84.11
2007	545.32	210.99	11.10	144.64	756.31	155.73	438.49	169.65	8.92	116.30	608.14	125.22
2008	655.20	218.60	12.97	99.31	873.80	112.28	497.51	165.99	9.85	75.41	663.50	85.26
2009	774.05	201.74	13.90	75.05	975.79	88.95	591.95	154.28	10.63	57.39	746.23	68.02
2010	967.83	268.73	21.29	82.61	1236.56	103.90	716.53	198.96	15.77	61.16	915.49	76.92
2011	1237.81	239.64	16.24	77.43	1477.45	93.67	869.53	168.34	11.41	54.39	1037.87	65.80
2012	1491.49	319.18	23.83	82.62	1810.67	106.46	1021.17	218.53	16.32	56.57	1239.70	72.89
2013	1734.37	367.13	31.26	66.22	2101.49	97.48	1157.31	244.98	20.86	44.19	1402.29	65.05
2014	1922.15	316.53	46.71	71.62	2238.69	118.32	1257.46	207.08	30.56	46.85	1464.54	77.41
2015	2219.66	335.57	63.31	84.67	2555.23	147.97	1432.03	216.50	40.84	54.62	1648.52	95.47

## 3 Results and discussion

In this section, we first analyze the models proposed in Sec. 2 with the accumulated expenditure data for technology innovation of China’s high-tech industry (1995~2015), Then, we use the criteria proposed in Sec. 3.7, i.e., MAPE, MAE, and RMSE, to compare the prediction accuracy of the three discretization methods.

### 3.1 Correlation analysis

The correlation analysis results for the six technology innovations in Table 5 show that the correlation coefficients of the six variables are very close to 1 and significant at the 0.01 level, implying that there is a clear correlation between the six variables, thus providing a foundation for studying the ecological relationships between variables.

**Table 5:** Correlation analysis for six technology innovation.

Pearson Correlation	RD	TR	II	DTP	FTA	TI	Mean	Std.
**RD**	1						2616.42	2959.92
**TR**	0.964**	1					1275.48	931.90
**II**	0.994**	0.980**	1				70.07	63.31
**DTP**	.903**	.984**	.932**	1			623.21	440.70
**FTA**	.998**	.979**	.997**	.929**	1		3891.91	3866.06
**TI**	.922**	.991**	.948**	.999**	.944**	1	693.28	500.25

Notes

** indicates that the parameter correlation is significant at the 0.01 level (2-tailed).

### 3.2 Regression analysis

According to the data in Table 4, we estimate the parameters of the Lotka-Volterra model using three discretization methods, namely, the Leslie method, the log-integral method, and the gray method. In the analysis process of ER1, RD was designated X_1_ and RD was designated X_2_. In the same way, DTP was designated X_1_ and FTA was designated X_2_ for ER2 and II was designated X_1_ and TI was designated X_2_ for ER3.

In the regression analysis of the Lotka-Volterra model with the Leslie method, we first estimate the parameter C_ij_ in Eqs (9) and (10) with the data in Table 4, substitute the values of C_ij_ into Eq (8) to obtain parameter b_ij_ of Eqs (3) and (4), and substitute the value of b_ij_ into Eq (5) to calculate parameter *α*_*ij*_ of Eqs (1) and (2). Table 6 shows the estimation and calculation results of each parameter of the Lotka-Volterra model with the Leslie method, and the findings show that the statistical significance of the estimated parameters is generally remained below 1%, with only a few parameters remaining below 5% according to the t-statistics of parameter C_ij_. The model fit is approximately 0.7 according to the R^2^ value, and the limit of F is below 0.01 according to the F value.

**Table 6:** Parameter estimation and calculation results with the Leslie method for the Lotka-Volterra model.

	ER1	ER2	ER3
Parameters	X_1_ = RD, X_2_ = TR	X_1_ = DTP, X_2_ = FTA	X_1_ = II, X_2_ = TI
**Eqs (9) and (10)**			
C_10_	0.5300839274***	0.7474831578***	0.6752635029***
C_11_	-0.0000728356***	-0.0016098349**	-0.0000202405**
C_12_	0.0003159672***	0.0002971904***	0.0002835352***
R^2^	0.8166	0.5741	0.6679
F	0.000	0.001	0.000
C_20_	0.6742858941***	0.6850355607***	0.6841250476***
C_21_	-0.0000587745***	-0.0009870160**	-0.0000206980**
C_22_	0.0002524581***	0.0003649598***	0.0003430436***
R^2^	0.7518	0.8452	0.8430
F	0.000	0.000	0.000
**Eqs (3) and (4)**			
b_10_	1.8864937196	1.3378227851	1.4809033743
b_11_	0.0001374040	0.0021536739	0.0000299743
b_12_	-0.0005960701	-0.0003975881	-0.0004198883
b_20_	1.4830504521	1.4597782325	1.4617210750
b_21_	0.0000871655	0.0014408245	0.0000302547
b_22_	-0.0003744082	-0.0005327604	-0.0005014341
**Eqs (1) and (2)**			
*α*_10_	0.6347199313	0.2910435054	0.3926522896
*α*_11_	0.0000983798	0.0018554485	0.0000244737
*α*_12_	-0.0004267798	-0.0003425330	-0.0003428341
*α*_20_	0.3941010829	0.3782845286	0.3796145599
*α*_21_	0.0000711148	0.0011854446	0.0000248746
*α*_22_	-0.0003054643	-0.0004383309	-0.0004122655

Note

*, ** and *** denote significance of p-values at the 0.1, 0.05 and 0.01 levels, respectively.

The parameters and model test results in Table 6 show that the parameters are significant according to the p-value of the t-test, the model has an extremely good fitting degree according to the values of R^2^ and the regression equation has obvious linear significance according to the p-value of the F-test.

### 3.3 Ecological relationship analysis

**Fig 2:**
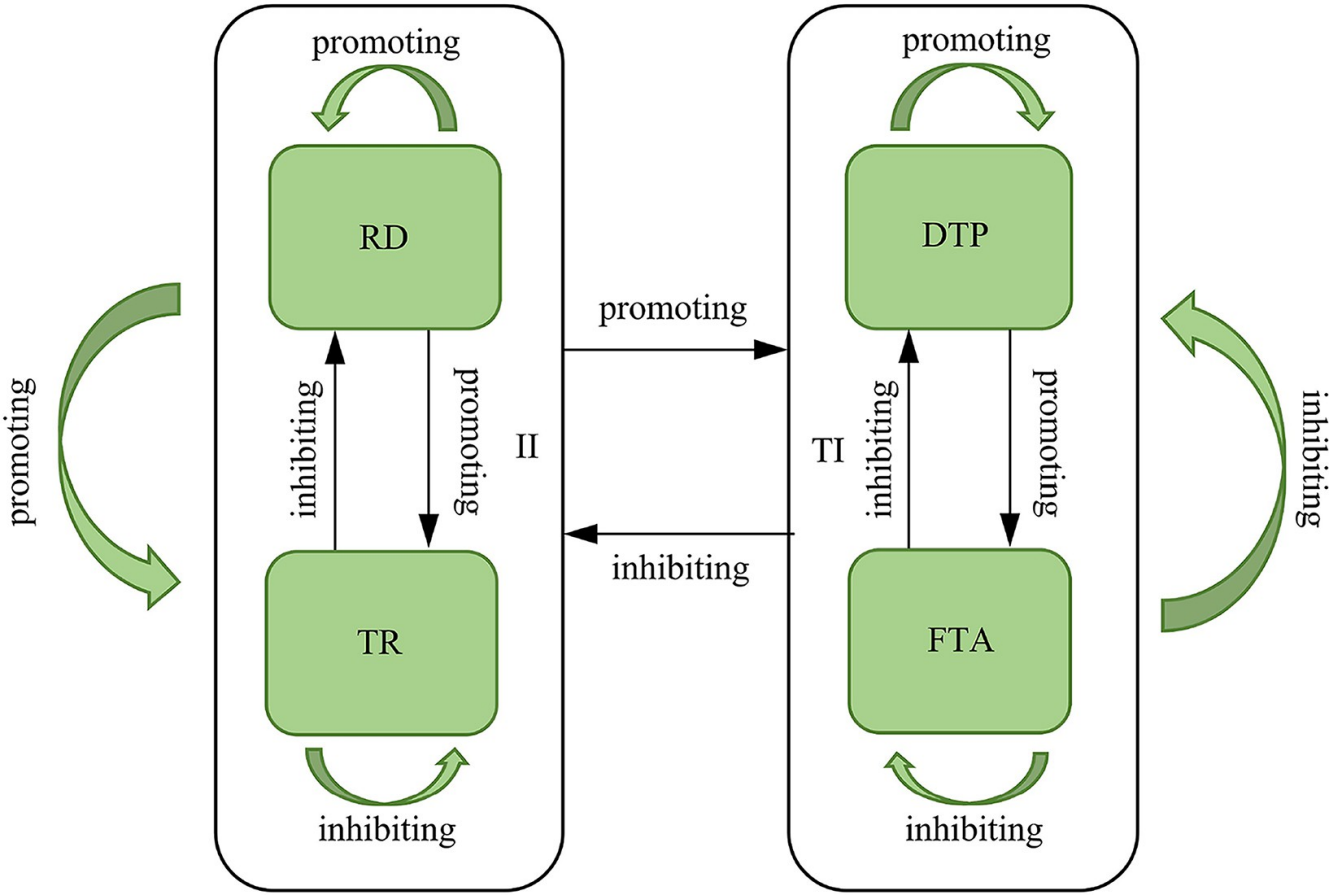
Ecological relationships of technology innovation.

Eqs (1) and (2) are based on the Lotka-Volterra model and contain all of the fundamental parameters that affect the growth rates of the six technology innovation scales. The ecological relationships among the six technology innovations can be determined through the parameter *α*_*ij*_ based on the intrinsic growth rate, niche capacity limitations, and interaction, as shown in Fig 2.

#### (1) Intrinsic growth rate

According to parameters *α*_10_ and *α*_20_ in Table 6, the intrinsic growth rate of six technology innovation species can be analyzed. The intrinsic growth rate parameters *α*_10_ and *α*_20_ of the six technology innovation species are all positive, indicating that the scale of these innovations is consistently growing from 1995 to 2015. In addition, the parameter *α*_10_ for RD is 0.6347199313, which is the maximum value among the six intrinsic growth rate parameters, thus implying that the average growth rate of RD is the fastest, while the parameter *α*_10_ of DTP is 0.2910435054, which is the minimum value among the six intrinsic growth rate parameters, thus implying that the average growth rate of DTP is the slowest. The results of the analysis are consistent with the actual data in Table 4, validating the applicability of the Lotka-Volterra model for analyzing technology innovation.

#### (2) Niche capacity limitations

According to parameters *α*_11_ and *α*_22_ in Table 6, the niche capacity limitations of the six technology innovation species can be examined. The values of Parameter *α*_11_ for RD, DTP and II are equal to 0.0000983798, 0.0018554485, and 0.0000244737, respectively, which are all positive. The regression results indicate that the niche capacity has a promoting effect on RD, DTP, and II. In addition, the value of DTP is the maximum and that for II is the smallest. Parameter *α*_22_ values for TR, FTA and TI are equal to -0.0003054643, -0.0004383309, and -0.0004122655, respectively, which are all negative. The regression results demonstrate that the niche capacity has an inhibitory effect on TR, FTA, and TI. The value of FTA is the maximum and that for TR is the smallest. These analytical results may be interpreted as follows. Although RD, DTP, and II, are the core power and significant for the sustainable development of the Chinese high-tech industry, the scale of these innovations is not sufficient to forcefully transform and quickly upgrade the Chinese high-tech industry. For the technology innovation system, niche capacity has a promoting effect on RD, DTP, and II. However, TR, FTA, and TI are not core power of the Chinese high-tech industry and represent supplements for RD, DTP, and II, respectively. The scales of TR, FTA, and TI are large enough to supplement RD, DTP, and II. Under the context of insufficient RD, DTP, and II and the systematic view of technology innovation, it is reasonable that niche capacity will have an inhibitory effect on TR, FTA, and TI.

#### (3) Interaction

Based on the regression results, the interaction parameters *α*_12_ and *α*_21_ in Table 6 and the type of the ecological relationships in Table 1, it is possible to examine the interaction between the six technology innovation species. For RD and TR (ER1), the parameter *α*_12_ is -0.0004267798, which is negative for RD, meaning that TR inhibits the subsequent scale of RD, while parameter *α*_21_ is 0.0000711148, which is positive for TR, implying that RD promotes the subsequent scale of TR. These analytical results demonstrate that RD is a prey species and TR is a predator species, with RD serving as direct food for TR.

For DTP and FTA (ER2), the parameter *α*_12_ is -0.0003425330, which is negative for DTP, implying that FTA inhibits the subsequent scale of DTP, while the parameter *α*_21_ is 0.0011854446 and positive for DTP, meaning that FTA promotes the subsequent scale of DTP. These analytical results demonstrate that DTP is a prey species and FTA is a predator species, with DTP serving as direct food for FTA.

For II and TI (ER3), the parameter *α*_12_ is -0.0003428341, which is negative for II, implying that TI inhibits the subsequent scale of II, while the parameter *α*_21_ is 0.0000248746, which is positive for TI, implying that II promotes the subsequent scale of TI. These analytical results demonstrate that II is a prey species and TI is a predator species, with II serving as a direct food for TI.

The results of the above analysis indicate three conclusions. First, all six technology innovations increased from 1995 to 2015. Second, RD, DTP, and II promote themselves while TR, FTA, and TI inhibit themselves. Third, the ecological relationships between RD and TR, DTP and FTA, and II and TI are all prey-predator relationships. Therefore, it can be seen that the population size change of each species of technology innovation is not only influenced by itself, but also by the number of other species, and the study of the relationship between the components of technology innovation from the ecological perspective is consistent with the need for technology innovation.

### 3.4 Evolutionary trend analysis

For RD and TR (ER1), by substituting parameter *α*_*ij*_ of RD in Table 6 into Eq (17), we can obtain Eq (21), and by substituting the parameter *α*_*ij*_ of TR in Table 6 into Eq (18), we can obtain Eq (22),

$$L_{1}:0.6347199313+0.0000983798RD(t)-0.0004267798TR(t)=0,$$ (21)

$$L_{2}:0.3941010829+0.0000711148RD(t)-0.0003054643TR(t)=0.$$ (22)

**Fig 3:**
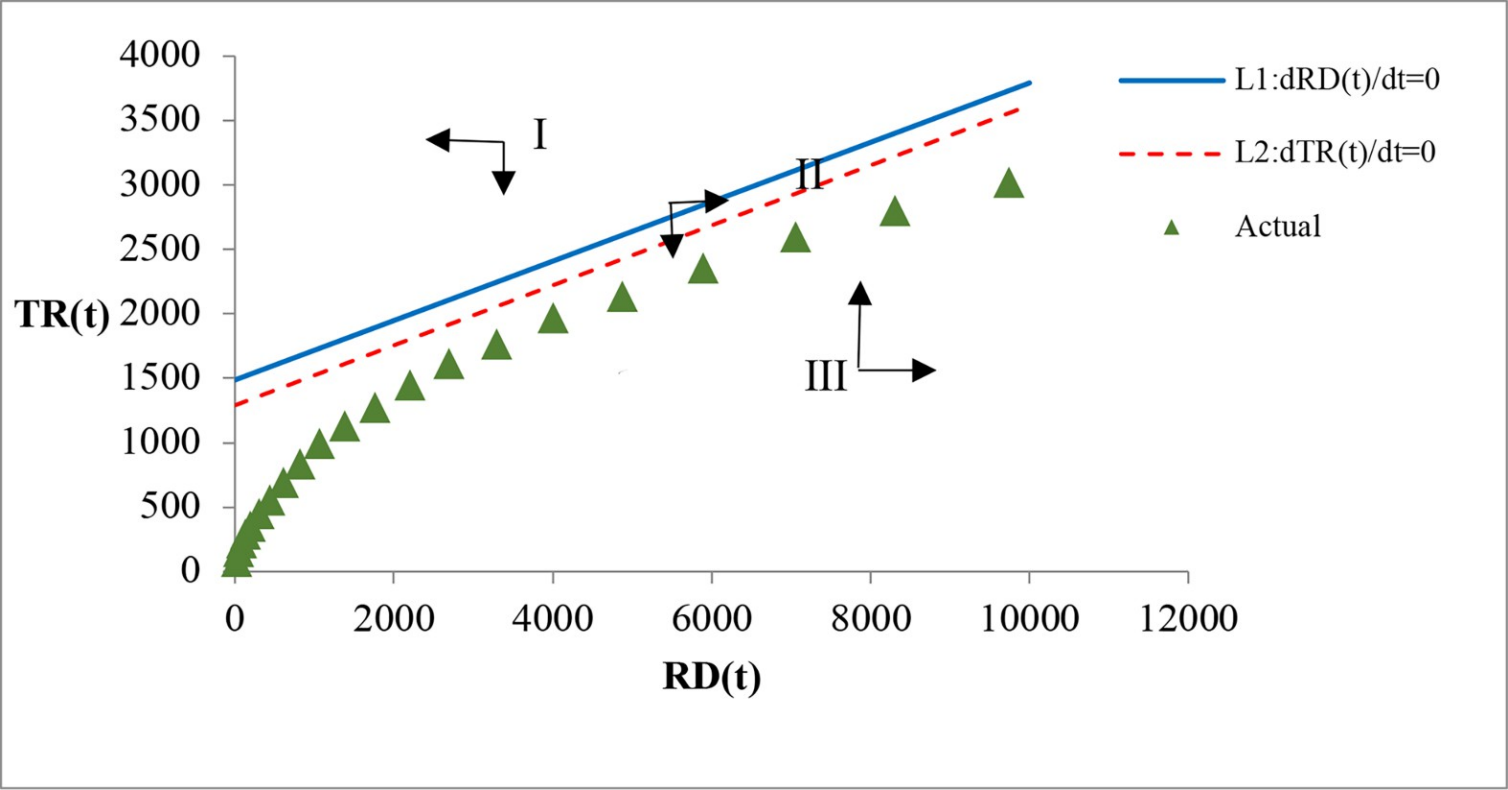
Partial magnified view of the evolutionary trend of RD and TR.

**Fig 4:**
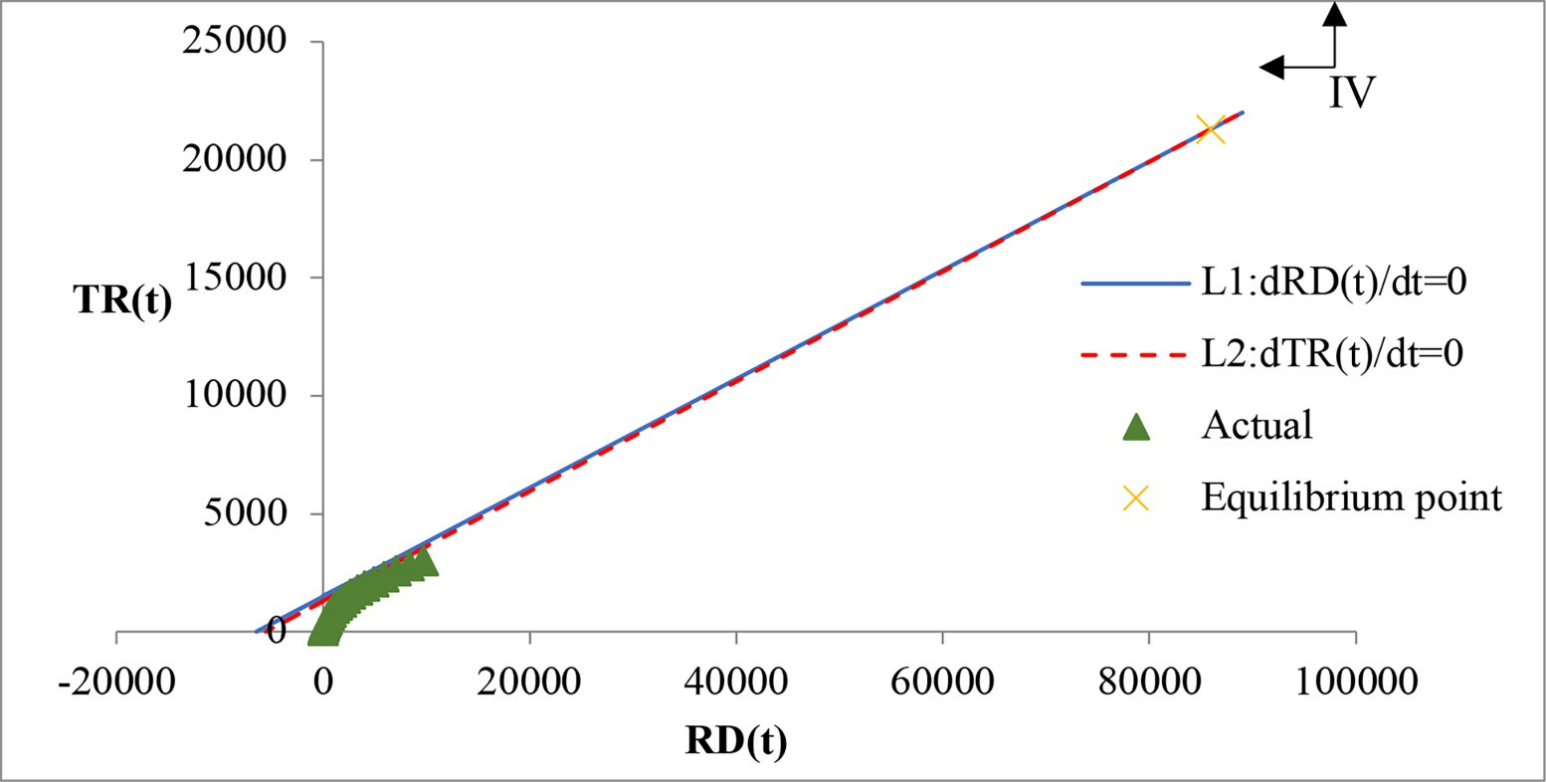
Overall view of the evolutionary trend of RD and TR.

Eqs (21) and (22) are two linear functions for the evolutionary trend analysis of RD and TR, as shown in Figs 3 and 4, where Fig 3 presents a magnified view of the lower left part of Fig 4. *L*_1_ represents the linear equation *dRD*(*t*)/*dt* = 0, and *L*_2_ represents the linear equation *dTR*(*t*)/*dt* = 0. The accumulative actual data of RD and TR are shown in Table 4. Since both RD and TR are greater than zero, only the region of the first quadrant is of practical interest for the analysis of the evolutionary trend of RD and TR. *L*_1_ and *L*_2_ intersect in the first quadrant, indicating an equilibrium point for RD and TR. In addition, *L*_1_ and *L*_2_ divide the first quadrant into four regions. For any point in region I, we can obtain the following inequalities (23) and (24):

$$L_{1}:0.6347199313+0.0000983798RD(t)-0.0004267798TR(t)<0,$$ (23)

$$L_{2}:0.3941010829+0.0000711148RD(t)-0.0003054643TR(t)<0.$$ (24)

In Eqs (23) and (24) show that, *dRD*(*t*)/*dt*<0 and *dTR*(*t*)/*dt*<0 according to Eqs (15) and (16). Therefore, in region I, RD and TR will decrease. Similarly, in region II, *dRD*(*t*)/*dt*>0 and *dTR*(*t*)/*dt*<0, indicating that RD will increase while TR will decrease. In region III, *dRD*(*t*)/*dt*>0 and *dTR*(*t*)/*dt*<0, indicating that RD and TR will increase. To the right of the equilibrium point, to the left of L1, and the right of L2 is region IV, where *dRD*(*t*)/*dt*<0 and *dTR*(*t*)/*dt*>0, indicating that RD will decrease and TR will increase.

Similarly, we can obtain two linear functions (25) and (26) for DTP and FTA (ER2):

$$L_{1}:0.2910435054+0.0018554485DTP(t)-0.0003425330FTA(t)=0,$$ (25)

$$L_{2}:0.3782845286+0.0011854446DTP(t)-0.0004383309FTA(t)=0.$$ (26)

**Fig 5:**
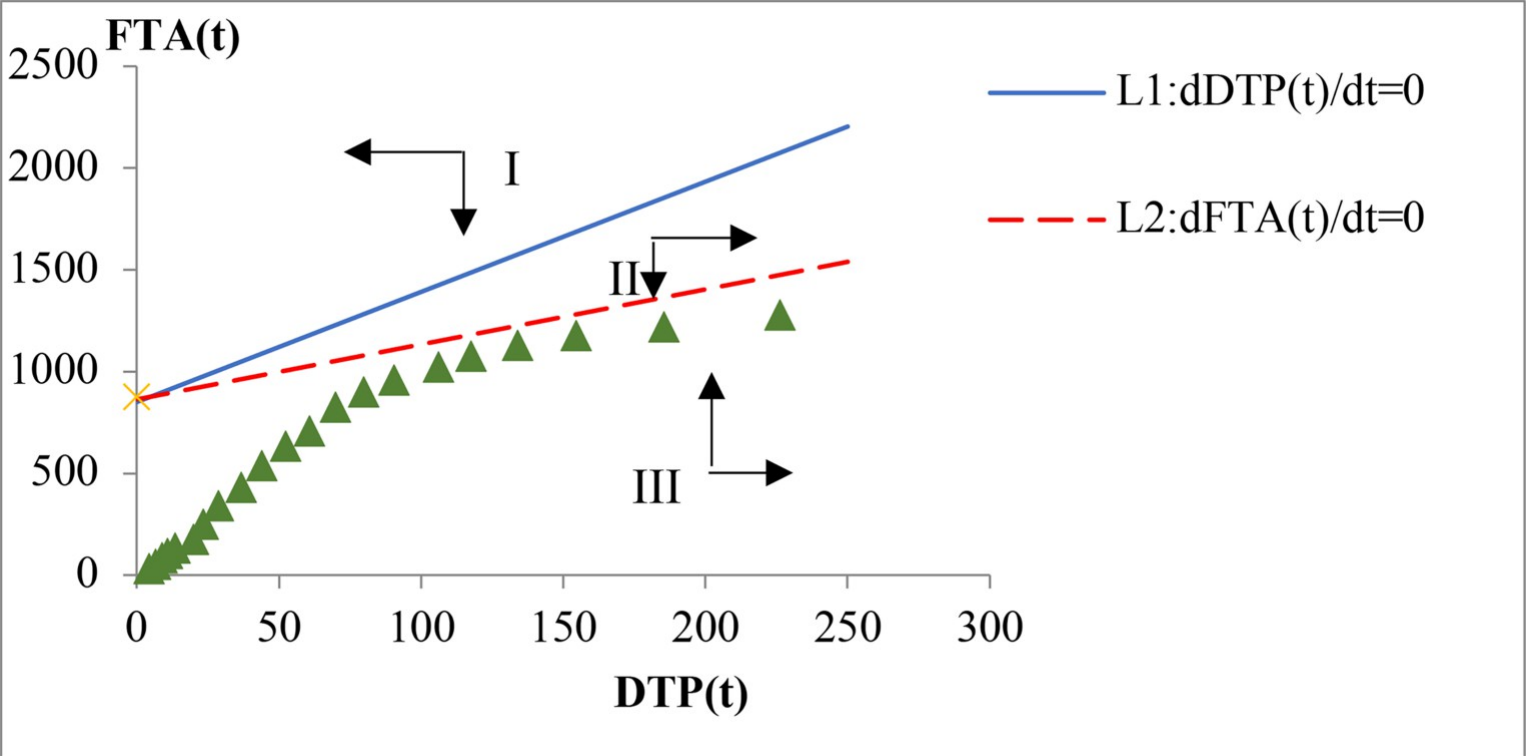
Evolutionary trend of DTP and FTA.

As shown in Fig 5, L1 and L2 intersect in the second quadrant, thus indicating that there is no equilibrium point for DTP and FTA. L1 and L2 divide the first quadrant into three regions. In region I, *dDTP*(*t*)/*dt*<0 and *dFTA*(*t*)/*dt*<0, indicating that DTP and FTA will decrease. In region II, *dDTP*(*t*)/*dt*>0 and *dFTA*(*t*)/*dt*<0, indicating that DTP will increase and FTA will decrease. In region III, *dDTP*(*t*)/*dt*>0 and *dFTA*(*t*)/*dt*>0, indicating that DTP and FTA will increase.

Similarly, we can obtain two linear functions (27) and (28) for II and TI (ER3):

$$L_{1}:0.3926522896+0.0000244737II(t)-0.0000244737TI(t)=0,$$ (27)

$$L_{2}:0.3796145599+0.0000248746II(t)-0.0004122655TI(t)=0.$$ (28)

**Fig 6:**
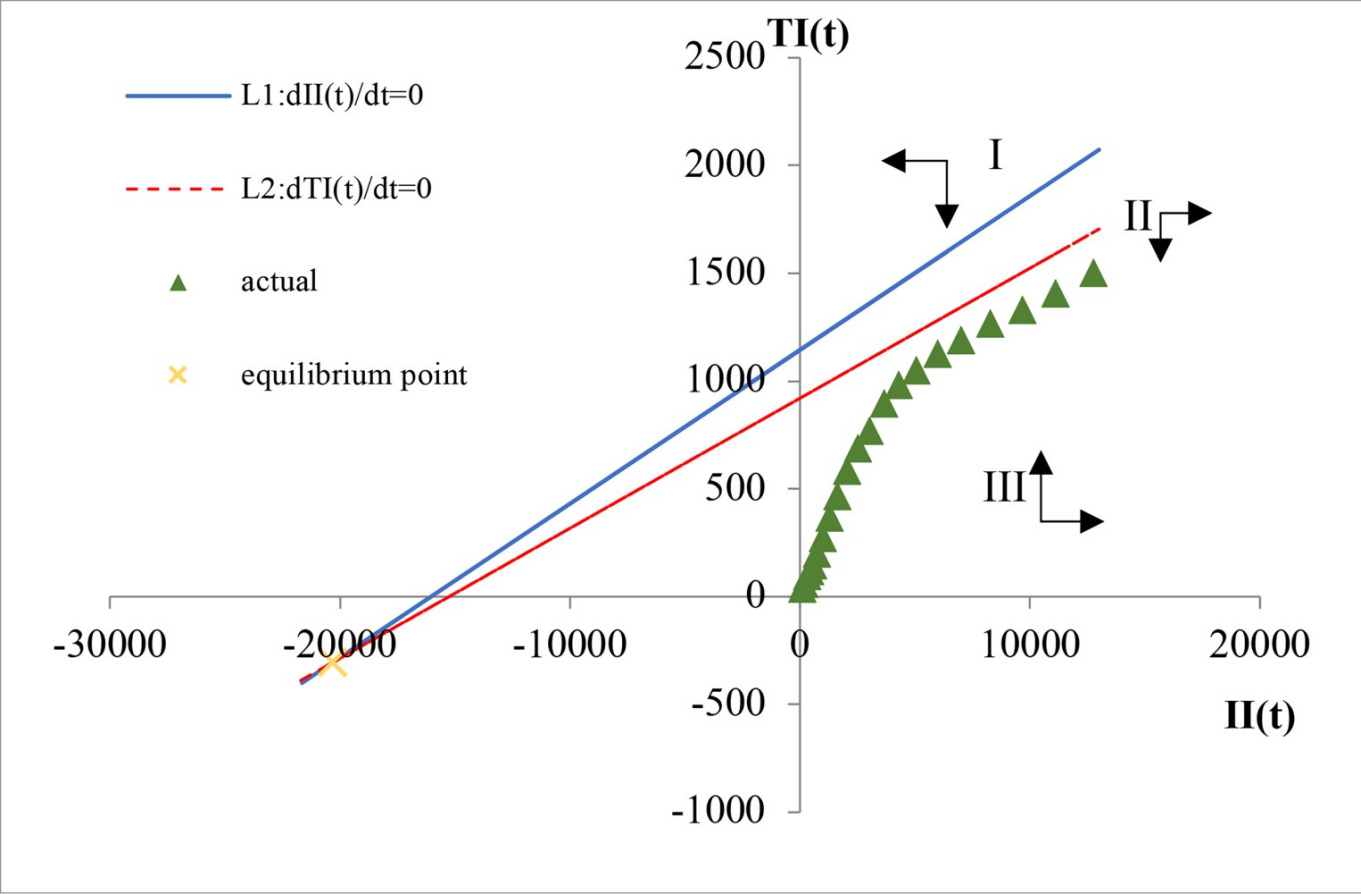
Evolutionary trend of II and TI.

As shown in Fig 6, L1 and L2 intersect in the third quadrant, indicating that there is no equilibrium point for II and TI. L1 and L2 also divide the first quadrant into three regions. In region I, *dII*(*t*)/*dt*<0 and *dTI*(*t*)/*dt*<0, indicating that II and TI will decrease. In region II, *dII*(*t*)/*dt*>0 and *dTI*(*t*)/*dt*<0, indicating that II will increase and TI will decrease. In region III, *dII*(*t*)/*dt*>0 and *dTI*(*t*)/*dt*>0, indicating that both II and TI will increase.

The results of the above analysis indicate three conclusions. First, an equilibrium point emerges between RD and TR, but its stability must be verified, while no equilibrium point emerges between DTP and FTA, II and TI. Second, RD, DTP, and II will decrease, while TR, FTA, and TI will increase.

Thus, it can be seen that opening the black box of technology innovation and considering each type of technology innovation as a species can provide a clearer study of the evolutionary trends of the components of technology innovation and provide worthy ideas for technology innovation research.

### 3.5 Equilibrium stability analysis

By substituting the values of the parameter *α*_*ij*_ in models ER1, ER2, and ER3 into Eq (19), we can obtain the equilibrium points of ER, as shown in Table 7. The actual economic data show that the data must be positive and *X*_1_>0, *X*_2_>0, according to which we can confirm that only ER1 has a realistic equilibrium point between RD and TR while ER2 and ER3 have no realistic equilibrium point, thus verifying the results in section 6.

**Table 7:** Equilibrium state analysis results.

Model	ER1	ER2	ER3
Equilibrium point	RD-TR	TD-TF	II-TI
$\dot{x_{1}}$	85963.89	-4.91	-20317.96
$\dot{x_{2}}$	21303.33	876.30	-305.11
Eigenvalues	1.77, 0.19	meaningless	meaningless
Stability	unstable	meaningless	meaningless

Next, the stability of the equilibrium point of the ER1 model is determined based on the eigenvalues of the Jacobian matrix. Substituting the values of the parameter *α*_*ij*_ and the equilibrium point of model ER1 into Eq (19), we can obtain the eigenvalues of the Jacobian matrix of model ER1, i.e., 1.77 and 0.19, according to which the real part of these eigenvalues is greater than zero. The eigenvalue does not satisfy the stability condition, thus implying that the stability of the equilibrium point of ER1 is unstable. Based on the above equilibrium analysis, we find that no equilibrium point appears between TD and TF and II and TI, and an unstable equilibrium point appears between RD and TR.

### 3.6 Robustness test

In this study, the regression analysis of the model was done again using the log-integral method and the gray method to verify the robustness of the results. Table 7 shows the results of the estimation and calculation of each parameter of the Lotka-Volterra model with the log-integral method, and the results of the study imply that the statistical significance of the estimated parameters is generally staying below 1%, a few parameters stay below 5%, and only one parameter stays above 10% according to the t-statistic of the parameter C_ij_. Based on the R^2^ value, the model fit is approximately 0.8, and the limit of F value is below 0.001. Table 8 shows the results of the estimation and calculation of each parameter of the Lotka-Volterra model with the gray method, and the results show that the statistical significance of the estimated parameters mostly stays below 1% level according to the t-statistic of the parameters C_ij,_ a few parameters stay below 5% or 10%, and only one parameter stays above 10%. The model fit is approximately 0.8 according to the R^2^ value, and the bounds of F are below 0.001 according to the F value.

**Table 8:** Parameter estimation and calculation results with the log-integral method for the Lotka-Volterra model.

Parameters	X_1_ = RD, X_2_ = TR	X_1_ = DTP, X_2_ = FTA	X_1_ = II, X_2_ = TI
Eqs (14) and (15)			
*α*_10_	0.6591910770***	0.3022873867***	0.4121569015***
*α*_11_	0.0001133476***	0.0017103457**	0.0000283351**
*α*_12_	-0.0004976134***	-0.0003533526***	-0.0004009004***
R^2^	0.853	0.757	0.779
F	0.000	0.001	0.000
*α*_20_	0.4098490374***	0.3863065808***	0.3870180408***
*α*_21_	0.0000748946***	0.0010445372	0.0000246257*
*α*_22_	-0.0003306169***	-0.0004374136***	-0.0004245225***
R^2^	0.825	0.894	0.894
F	0.000	0.000	0.000
**Eqs (3) and (4)**			
b_10_	1.9332278692	1.3529499906	1.5100713503
b_11_	0.0001604680	0.0019969953	0.0000350666
b_12_	-0.0007044797	-0.0004125736	-0.0004961407
b_20_	1.5065903291	1.4715357462	1.4725830576
b_21_	0.0000925728	0.0012749890	0.0000300701
b_22_	-0.0004086562	-0.0005339183	-0.0005183793

The results of the parameters and model tests in Tables 8 and 9 show that the parameters are significant according to the p-value of the t-test, that the model fits very well according to the value of R^2^, and that the regression equation is significantly linear according to the p-value of the F-test. Thus, the results show a high degree of robustness.

**Table 9:** Parameter estimation and calculation with the gray method for the Lotka-Volterra model.

Parameters	X_1_ = RD, X_2_ = TR	X_1_ = DTP, X_2_ = FTA	X_1_ = II, X_2_ = TI
Eqs (19) and (20)			
*α*_10_	0.6327161068***	0.2995285540***	0.4042741273***
*α*_11_	0.0001049995***	0.0016863689**	0.0000270985*
*α*_12_	-0.0004648945***	-0.0003480361***	-0.0003863434***
R^2^	0.867	0.759	
F	0.000	0.001	
*α*_20_	0.4025096694***	0.3812632229***	0.3820031752***
*α*_21_	0.0000724926***	0.0010076908	0.0000239030*
*α*_22_	-0.0003212874***	-0.0004279809***	-0.0004155873***
R^2^	0.832	0.897	0.788
F	0.000	0.000	0.000
**Eqs (3) and (4)**			
b_10_	1.8827173022	1.3492225720	1.4982145923
b_11_	0.0001464873	0.0019661501	0.0000333953
b_12_	-0.0006485854	-0.0004057779	-0.0004761174
b_20_	1.4955733864	1.4641329480	1.4652167375
b_21_	0.0000892535	0.0012267181	0.0000291099
b_22_	-0.0003955718	-0.0005210049	-0.0005061167

### 3.7 Comparison of the forecast ability levels

Substituting the actual data in Table 4 and parameter values of b_ij_ in Tables (6) to (8) obtained by the three discretization methods into Eqs (3) and (4), we can calculate the estimated values of the six technological innovations by the three discretization methods. Then, substituting the estimated values and the actual data into Eqs (11) ~ (14), we can obtain the values of MAPE, MAE, and RMSE for the six technological innovations by the three discretization methods, as shown in Table 10. Among the three discretization methods, the Leslie method has the smallest MAPE value and the same MAE and RMSE values for the six technological innovations, which means that the Leslie method is the most suitable for the Lotka-Volterra model among the three discretization methods. Therefore, this paper analyzes the ecological relationships, evolutionary trends, and equilibrium states of the six technological innovations using the estimated parameters of the Lotka-Volterra model with the Leslie method.

**Table 10:** MAPE, MAE, and RMSE.

	Discretization method	RD	TR	DTP	FTA	II	TI
**MAPE**	Leslie	10.22%	6.71%	3.68%	3.67%	9.44%	3.68%
	Log_int	26.02%	13.19%	5.54%	4.34%	11.11%	4.41%
	Gray	31.01%	13.40%	5.21%	3.90%	11.68%	4.72%
**MAE**	Leslie	157.81	55.24	1.08	9.48	155.74	10.65
	Log_int	728.69	149.99	2.44	12.85	276.28	14.40
	Gray	814.82	155.46	2.18	10.16	298.14	17.83
**RMSE**	Leslie	300.43	79.70	1.38	11.78	247.88	12.63
	Log_int	1364.01	261.54	3.60	17.60	513.36	22.85
	Gray	1450.42	276.24	3.16	15.28	588.28	26.51

## 4 Conclusions and policy implications

### 4.1 Conclusions

This study investigates the ecological relationships among technology innovations in China’s high-tech industry. Based on the Lotka-Volterra model and the data from 1995–2015 in the Statistical Yearbook of China’s High-Tech Industries, we derive interesting and important conclusions. First, the Leslie method is the best discretization method for the Lotka-Volterra model and the Lotka-Volterra model is suitable for describing and predicting the scale changes of six technology innovation species in Chinese high-tech industries. Second, the ecological relationships between RD and TR, DTP and FTA, II and TI are prey-predator relationships, and RD and DTP and II will be greatly reduced, while TR and TF and TI will be gradually increased. Third, there is no equilibrium point between DTP and FTA, II and TI, and an unstable equilibrium point between RD and TR.

### 4.2 Implications

Based on the above analysis, this study draws the following three policy implications:

1. The government should strengthen its support and subsidies for high-tech industries. While reducing R&D uncertainty and risk, the government should also focus on the implementation of anti-monopoly rules and strategies to prevent large downstream buyers from abusing market dominance and protect the innovation resources of enterprises, and create a good market competition environment for enterprise technology innovation.
2. The government should continue to promote globalization and introduce R&D and innovation foreign direct investment under local conditions. While improving the intellectual property protection system, the government should introduce high-quality foreign investment in a targeted manner, give full play to the role of foreign technology innovation in promoting the technological progress of domestic enterprises, and reasonably optimize the level of foreign-funded competition in different industries rationally to provide nourishment for improving local independent innovation capability.
3. High-tech enterprises should implement technology diversification strategy, and realize both collaborative and exploratory innovation. Enterprises should make full use of the advantageous knowledge resources of the members of the collaborative innovation network, give full play to the important organizational capabilities of product innovation, optimize the structure through technological diversification, and open up various links of resource integration.

### 4.3 Limitations

Two limitations should be taken into consideration in subsequent research. First of all, this study only examined the input data of technological innovation because it is difficult to obtain the output data of technological innovation, which can also indicate the development trend of various technological innovations. Moreover, we ignore the life periodicity of technology innovation of the high-tech industry. It would be meaningful to study the evolution of ecological relationships among different varieties of technology innovation during various periods of the life cycle.

## Supporting information

S1 Data (XLSX)

S1 Appendix (DOCX)
