# Supplementary material for: Analyzing the ecological relations of technology innovation of the Chinese high-tech industry based on the Lotka-Volterra model
Source: PLoS One. 2022 May 31;17(5):e0267033. doi: 10.1371/journal.pone.0267033 (PMC9154195; doi:10.1371/journal.pone.0267033)
Supplement: S1 Appendix — (DOCX) [file pone.0267033.s002.docx]

**Appendix 1**

**1. Log-integral method**

According to the Log-integral method [44], equations (1) and (2) can be transformed into equations (29) and (30):

$$\ln X_{1}\left( t+1 \right)-\ln X_{1}\left( t \right)\approx\alpha_{10}+\alpha_{11}\frac{X_{1}\left( t+1 \right)+X_{1}\left( t \right)}{2}+\alpha_{12}\frac{X_{2}\left( t+1 \right)+X_{2}\left( t \right)}{2}, (291)$$

$$\ln X_{2}\left( t+1 \right)-\ln X_{2}\left( t \right)\approx\alpha_{20}+\alpha_{21}\frac{X_{1}\left( t+1 \right)+X_{1}\left( t \right)}{2}+\alpha_{22}\frac{X_{2}\left( t+1 \right)+X_{2}\left( t \right)}{2}. (30)$$

Let

$$Y_{i}=\ln X_{i}\left( t+1 \right)-\ln X_{i}\left( t \right), Z_{i}=\frac{X_{i}\left( t+1 \right)+X_{i}\left( t \right)}{2}, i=1,2. (31)$$

Equations (29) and (30) can be transformed into equations (32) and (33):

$$Y_{1}=\alpha_{10}+\alpha_{11}Z_{1}+\alpha_{12}Z_{2}, (32)$$

$$Y_{2}=\alpha_{20}+\alpha_{21}Z_{1}+\alpha_{22}Z_{2}. (33)$$

Using the linear least square method, we can estimate the parameters in equations (32) and (33).

**2. Gray method**

According to the gray method [45-48], equations (1) and (2) can be transformed into equations (34) and (35):

$$\frac{2(X_{1}\left( t+1 \right)-X_{1}(t))}{X_{1}\left( t+1 \right)+X_{1}(t)}=\alpha_{10}+\alpha_{11}\frac{X_{1}\left( t+1 \right)+X_{1}(t)}{2}+\alpha_{12}\frac{X_{2}\left( t+1 \right)+X_{2}(t)}{2}, (34)$$

$$\frac{2(X_{2}\left( t+1 \right)-X_{2}(t))}{X_{2}\left( t+1 \right)+X_{2}(t)}=\alpha_{20}+\alpha_{21}\frac{X_{1}\left( t+1 \right)+X_{1}(t)}{2}+\alpha_{22}\frac{X_{2}\left( t+1 \right)+X_{2}(t)}{2}. (35)$$

Let

$$Y_{i}\left( t \right)=\frac{2\left( X_{i}\left( t+1 \right)-X_{i}\left( t \right) \right)}{X_{i}\left( t+1 \right)+X_{i}\left( t \right)},Z_{i}\left( t \right)=\frac{X_{i}\left( t+1 \right)+X_{i}(t)}{2},i=1,2. (36)$$

Then, equations (34) and (35) can be converted into linear relationships (37) and (38):

$$Y_{1}\left( t \right)=\alpha_{10}+\alpha_{11}Z_{1}\left( t \right)+\alpha_{12}Z_{2}\left( t \right), (37)$$

$$Y_{2}\left( t \right)=\alpha_{20}+\alpha_{21}Z_{1}\left( t \right)+\alpha_{22}Z_{2}\left( t \right). (38)$$

Using linear regression, we can estimate the parameters in equations (37) and (38).
